# THE EFFECT OF ONLINE IN-GAME INTERGENERATIONAL CONTACT ON AGEISM TOWARD THE OPPOSITE GENERATION

**DOI:** 10.1093/geroni/igad104.0250

**Published:** 2023-12-21

## Abstract

Previous research indicated that online games represent a unique context of intergroup contact but may enhance prejudice toward outgroup players during gameplay. Despite the growing number of older gamers and increasing opportunities of online in-game intergenerational contact, few study have focused on the online in-game intergenerational contact and its effect on ageism. To address such research gap, we designed an online quiz game by manipulating in-game intergenerational contact type (competition vs. cooperation) and game outcome (win vs. loss). 148 young participants (Mage = 22.71, SD = 2.88) and 155 older participants (Mage = 65.4, SD = 4.23) were recruited and randomly assigned into the four experimental conditions to play with a virtual game partner of opposite generation. Positive and negative dimensions of age-stereotype perception, individual’s cooperative orientation, and potential covariates were measured. Results showed significant intergenerational contact type × outcome × cooperative orientation effects on the perception of negative old-age-stereotype among younger participants and positive young-age-stereotype among older participants. Younger participants showed significant more negative old-age-stereotypes under competition loss (vs. win) condition, but older participants showed significant more positive young-age-stereotypes under cooperation loss (vs. win) condition. The findings indicated the negative impact of intergenerational competition among younger participants in the online game context, and also suggested the internalized self-ageism among older participants. Moreover, the boundary condition of personal factors revealed, that the effects of in-game intergenerational contact were only significant among those of lower (vs. higher) cooperative orientation. Our study also offered practical implications on designing gamified intergenerational-based intervention.

